# Excess sulfur and Fe elements drive changes in soil and vegetation at abandoned coal gangues, Guizhou China

**DOI:** 10.1038/s41598-020-67311-z

**Published:** 2020-06-26

**Authors:** Junyong Ma, Zhanjun Quan, Yibo Sun, Jiaqiang Du, Bo Liu

**Affiliations:** State Key Laboratory of Environmental Criteria and Risk Assessment, Chinese Research Academy of Environmental Sciences, Beijing, 100012 China

**Keywords:** Biochemistry, Chemical biology, Plant sciences, Biogeochemistry, Solid Earth sciences

## Abstract

Coal gangue piles accumulate outside mines and can persist for years, negatively impacting the regional environment. To determine the main cause of soil pollution at coal gangues, several coal gangues in Guizhou Province, China that had undergone natural recovery via native plants for 8 years were investigated in summer 2019. Three plots (2 m × 2 m) from the coal gangue area were selected for the treatment (GP). Control plots that were 100 m away from GP were also investigated in contrast (CK-near). In addition, plots from forest, farmland and lake land that were far from GP and largely undisturbed were also investigated as more extreme contrasts (CK-far). A series of soil indicators that can be affected by coal-gangue, such as heavy metals (Mn, Cr, Cd, Ni, Zn, Cu, Pb), As, pH, cation exchange capacity (CEC), sulfur (S) and iron (Fe), were tested for in the plots. Plant species, coverage and height were also analyzed to uncover biodiversity and dominant species information. The results suggested that coal gangue significantly influences soil S, pH and plant species after 8 years of natural recovery. The CK-far plots contained relatively low soil sulfur content, normal pH (close to 7) and abundant plant biodiversity. Generally, pH related positively with both the Patrick (R = 0.79, n = 22, p < 0.001) and Shannon indices (R = 0.67, n = 22, p < 0.001); the soil S related negatively with both the Patrick (R = 0.85, n = 22, p < 0.001) and Shannon indices (R = − 0.79, n = 22, p < 0.001). S content was highest (S = 1.0%) in GP plots, was lower in CK-near plots (S = 0.3%) and was the lowest of all in the plots distant from the coal mine (S = 0.1%, CK-far). S content was negatively correlated with pH. Soil pH decreased significantly, from 7.0 in CK-far, to 5.9 in CK-near, to 4.2 in GP. Soil Fe was 3.4 times higher in GP and CK-near than in CK-far. The excess sulfur and Fe elements and the acidified soil drove changes in soil and vegetation in the coal gangue areas. After 8 years of natural recovery, only a few plants, like *Miscanthus floridulus*, were able to live near the coal gangue in the area where the soil was still acidic and high in S and Fe.

## Introduction

Coal mining is indispensable due to the widespread use of coal, and gangue is the accumulated solid waste byproduct that persists in mined lands, even after the mines have been abandoned^1^. In the past 2 decades the growth of the coal mining industry has resulted in large amounts of accumulated coal gangue in China^2,3^. Guizhou's annual coal output reached 139.17 million tons in 2018, ranking the fifth in China. Due to the extensive resource-based production mode, the coal industries here have caused serious environmental pollution and resource waste. Coal gangue contain various harmful components, such as heavy metals like Pb, Cd, Cr, Cu, Zn, Hg, Ni, Mn and As, which accumulate in nearby soils^4,5^.

Due to natural processes like efflorescence, sun exposure, rain leaching, the release of heavy metals and other elements, gangue readily affects the surrounding environment, including soil^1,6^, water^7^ and plants^8,9,10^. When exposed to atmospheric oxygen conditions, waste water that leaches from coal gangue may produce acidic drainage. This is due to the oxidation of sulfide minerals, which is also associated with a variety of toxic heavy metal elements. Thus, the drainage is a serious environmental pollutant^11,12^. At a large number of abandoned coal mining areas, coal mining waste and discharge pollutes the surrounding soil and water, thereby affecting agricultural land and even human populations. Heavy-metal polluted soil is regarded as a major threat to human and environmental health^13,14,15^, and heavy metals are quite common in the soil around coal gangue dumps^3,16,17^. Ao and Huang’s research (2005)^18^ conducted in the Wuda mining gangue dump of Inner Mongolia found that the environment had been heavily polluted by the low soil pH value and the high concentration of SO_4_^2–^ in the surface water around the gangue dump.

Many previous studies on coal gangue have mainly focused on leaching^19,20^. For example, Querol^3^ indicated that the leaching of weathered coal gangue and gas vent condensates may give rise to relatively more environmental problems than the leaching of trace elements from fresh coal gangue.

Guizhou province has plentiful coal resources and also relatively high vegetation coverage because the local government pledged in 2015 to achieve 60% forest coverage by 2020^21^. In fact, because Guizhou province has recently started to prioritize ecological development, many coal mines in Huaxi District were closed in the 2010s. Near the entrances and within a small radius of these abandoned mines, some gangues are still piled up. Thus, these coal gangue dumps can be paired with the undisturbed lands nearby to compare and study how residual coal gangue dumps affect the surrounding soil and plants. Soil contains a mass of chemical information, and plants are sensitive indicators of the environment, so studying these two elements offers a fairly holistic picture.

After studying the impact of a gully-type coal gangue dump in the Daliuta mine area of Shaanxi, China, Liu^22^ suggested that covering coal gangues with a 30 cm-thick loess layer and planting *Alfalfa* and *Artemisia ordosica* can significantly improve the ecological environment, even raising the vegetation coverage from 10% to about 65% in 5 years. Selecting suitable species for ecological restoration has been one of the important and practical issues studied in ecological restoration research for coal gangue fields. Here, we opted to study the coal gangue piles and the surrounding control areas in Huaxi district, Guizhou province, a region with relatively consistent conditions, by measuring and investigating the soil and vegetation species.

In doing so, we aimed to answer the following questions: (1) What is the short-term state of natural ecological recovery at abandoned coal mines in Guizhou province, China? (2) What is the main cause of soil pollution at these sites? and (3) How can native plants improve the condition of a habitat that has been polluted by gangue? Three coal mines in Guizhou Province that had roughly equivalent annual outputs before they were closed in 2011 were selected for this study. In July 2019, the vegetative species and soil composition, especially heavy metal elements, were recorded and sampled at each coal gangue and analysed. About 100 m away from each gangue, the same data was recorded at plots where the vegetation appeared largely unaffected by the mining residue. To draw a comparison to plots with even less of a gangue influence, we also selected distant undisturbed plots to reflect natural forest, agriculture and lake ecosystems and collected the same plant and soil data there.

## Results

### Soil

**Figure 1:**
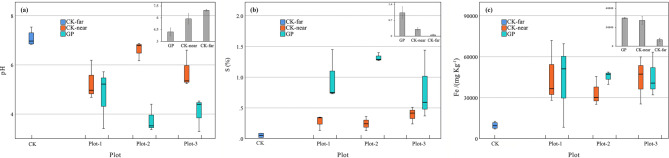
Difference in soil pH (**a**), S (**b**) and Fe levels (**c**) between abandoned and undisturbed plots. GP: plots in the gangue; CK-near: plots 100 m from the gangue; CK-far: plots distant from the gangue. Three plots were demarcated and investigated for each type.

Generally, the soil was relatively acidic in the study area. The average soil pH of CK-near plots was 5.9 ± 0.69. The GP contained even more acidic soil, with a soil pH averaging 4.2 ± 0.53. This (both GP and CK-near) pH was 28.3% lower than the CK-far plots, a difference that was significant at p < 0.001 (Table 1). The pH value of CK-far was 7.0 ± 0.12 (Fig. 1a).

**Table 1:** One-way Anova for soil properties between treatments.

Soil properties	F	p
pH	20.815	**< 0.001**
Fe	7.321	**0.004**
S	22.457	**< 0.001**
CEC	1.153	0.337
As	1.812	0.191
Cd	4.141	**0.032**
Cu	0.192	0.827
Pb	1.438	0.262
Ni	2.222	0.136
Zn	3.675	**0.045**
Mn	1.002	0.387
Cr	0.246	0.784
Hg	0.105	0.901
Patrick	18.670	**< 0.001**
Shannon	27.744	**< 0.001**
Pielou	5.495	**0.013**

Bold values indicate p < 0.05.

The soil sulfur (S) was detected to be much higher in the soil of GP than CK-near and CK-far plots. CK-near soil contained 0.1 ± 0.06% sulfur, and GP had 1.0 ± 0.21%. The latter level was 8.5 times higher than CK-far (0.05 ± 0.033%), which was significant at p < 0.001 (Fig. 1b).

GP and their CK-far distant counterparts did not differ significantly in soil Fe content. High Fe levels were detected in the soils of both GP (44.3 ± 6.01 g kg^−1^) and CK-near (43.3 ± 22.02 g kg^−1^) plots. Fe levels were significantly higher in disturbed areas (both GP and CK-near) than undisturbed areas (CK-far, 10.3 ± 2.34 g kg^−1^) (Fig. 1c). Soil Mn levels were 215.1 ± 137.43 mg kg^−1^ in GP, 418.4 ± 309.51 mg kg^−1^ in CK-near and 462.8 ± 183.76 mg kg^−1^ in CK-far (Table 2).

**Table 2:** Soil heavy metals content distribute between treatments.

	CEC (cmol/kg)	As (mg/kg)	Cd (mg/kg)	Cu (mg/kg)	Pb (mg/kg)	Ni (mg/kg)	Zn (mg/kg)	Mn (mg/kg)	Cr (mg/kg)	Hg (mg/kg)
GP	15.2 ± 1.92	8.4 ± 1.98	0.8 ± 0.14	61 ± 8.93	14.1 ± 3.93	13.3 ± 3.92	40.5 ± 22.22	215.1 ± 137.43	83.4 ± 10.06	0.01 ± 0.0015
CK-near	13 ± 3.71	7.9 ± 1.46	0.7 ± 0.21	63.2 ± 7.97	20.2 ± 2.22	17.6 ± 7.68	54.7 ± 18.07	418.4 ± 309.51	86 ± 0.46	0.01 ± 0.001
CK-far	12.9 ± 2.19	10.5 ± 2.02	1.3 ± 0.74	57.2 ± 32.99	19.3 ± 2.3	30 ± 25.81	95.2 ± 49.29	462.8 ± 183.76	81.8 ± 16.83	0.01 ± 0.0033

### Plants

**Figure 2:**
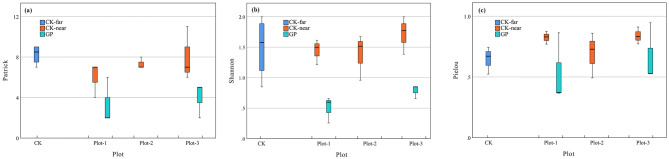
Differences in plant biodiversity indices of Patrick (**a**), Shannon (**b**) and Pielou (**c**) between abandoned and undisturbed plots. GP: plots in the gangue; CK-near: plots 100 m from the gangue; CK-far: plots distant from the gangue. Three plots were demarcated and investigated for each type.

The plots with the least interference contained the highest plant species richness, as indicated by the Species richness index. On average, plant species richness differed significantly between GP (about 3 species) and the control plots, with CK-far having slightly higher species richness than CK-near (Fig. 2a). The Shannon index, another species diversity index, also showed that the control plots had significantly (p < 0.001) higher Shannon index, with 0.4 ± 0.40 for GP, 1.5 ± 0.18 for CK-near and 1.3 ± 0.28 for CK-far (Fig. 2b). Lastly, a significantly higher species evenness (p = 0.008) was measured in CK-near (0.8 ± 0.08) than in GP (0.4 ± 0.35). Species evenness was 0.7 ± 0.11 in CK-far plots (Fig. 2c).

*Miscanthus floridulus* was the dominant species in the disturbed area by abundance, percent ground coverage and height. *H. punctate* appeared in the CK-near plots. Although both *M. floridulus* and *H. punctate* plants existed in the CK-far group, neither was the dominant species. The plot abundance differed significantly between CK-far and the other two plots (CK-near and GP), with 68 ± 34 plants at GP, 101 ± 55 in CK-near and 222 ± 22 in CK-far (Table 3).

**Table 3:** Basic geographic and vegetation information.

Plots treatments	Geographical coordinates	Altitude (m)	Treatment	Order number	Family number	Plant total number	Cover (%)	Height (cm)	Dominant species
Plot-1	106°34′55″E, 26°29′21″N	1,147	GP-1	5	7	291	75	26	*Miscanthus floridulus*
			GP-2	2	2	14	100	85	*Miscanthus floridulus*
			GP-3	1	1	5	80	50	*Miscanthus floridulus*
			CK-near-1	6	7	297	70	42	*Artemisia argyi*
			CK-near-2	7	8	93	50	82.5	*Hypolepis punctata*
			CK-near-3	6	7	112	60	43	*Hypolepis punctata*
Plot-2	106°34′04″E, 26°31′33″N	1,194	GP-1	3	3	130	100	50	*Miscanthus floridulus*
			GP-2	3	3	13	60	42	*Miscanthus floridulus*
			GP-3	4	5	52	80	51.3	*Miscanthus floridulus*
			CK-near-1	5	7	33	40	59.3	*Artemisia argyi*
			CK-near-2	6	7	51	30	39	*Humulus scandens*
			CK-near-3	6	8	202	45	65.6	*Clinopodium chinense*
Plot-3	106°33′44″E, 26°31′23″N	1,258	GP-1	3	5	36	46	47	*Miscanthus floridulus*
			GP-2	3	4	54	34	54	*Miscanthus floridulus*
			GP-3	2	2	34	56	90	*Miscanthus floridulus*
			CK-near-1	6	6	33	65	43.1	*Hypolepis punctata*
			CK-near-2	10	12	80	69	46.8	*Miscanthus floridulus*
			CK-near-3	4	7	33	81	80	*Salsolacollina Pall*
CK-far	106°35′56″N, 26°30′03″N	1,122	CK-far-1	6	7	243	95	100.7	*Paspalum paspaloides*
	106°33′59″N, 26°29′39″N	1,117	CK-far-2	4	9	247	90	86.9	*Pogonatherum crinitum*
	106°33′55″N, 26°31′52″N	1,120	CK-far-3	7	9	212	87	83.6	*Salsolacollina Pall*
	106°34′49″N, 26°32′33″N	1,137	CK-far-4	5	6	198	99	85.2	*Pogonatherum crinitum*

### Relationship between soil and plants

The soil S and pH have a significant effect on the distribution of biodiversity. pH related positively with both the Patrick (R = 0.79, n = 22, p < 0.001) and Shannon indices (R = 0.67, n = 22, p < 0.001); S related negatively with both the Patrick (R = 0.85, n = 22, p < 0.001) and Shannon indices (R = − 0.79, n = 22, p < 0.001). The CK-far plots showed relatively low soil sulfur content, high pH (close to 7) and abundant plant biodiversity.

## Discussion

The baseline findings in the studied plots indicated that it was relatively normal for these plots to have acidic soil, with GP having a pH of 4.2 and CK-far having a pH of 5.9. Soil Fe levels were higher surrounding the abandoned coal mines (GP and CK-near) and much lower in the distant CK-far plots. Similarly, soil S, was high in the abandoned coal mine plots (GP) and low in both control plots (CK-near and CK-far). The plants were greatly affected by the coal gangue in both species richness and abundance. In the area of or near former coal mines (GP and CK-near), plants like *M. floridulus*^23^ and *H. punctate*^24^, which live readily in moist areas, and show characteristics of drought-tolerance and acidic soil-resistance, became the dominant species.

### Soil characteristics

In this study, pH was significantly affected by coal gangue presence, as shown by the relatively normal pH in the CK-far plots. Acidic materials may leach out of coal gangue, thereby causing the surrounding soil (within 100 m) to maintain an acidic pH, even 8 years after abandonment and the beginnings of natural ecological restoration. Acidic soil research conducted in the northeastern United States and western Europe has revealed that when external proton loading exceeds internal proton loading, this could result in soil acidification via aluminum dissolution and sulphate retention^25^. During hydrolysis of Al_2_(SO_4_)_3_ (1) and FeSO_4_ (2) and the oxidation of pyrite (3), protons are absorbed into the soil. This can lead to soil acidification or even extreme acidification. In pyrite regions, soil pH can reach lows of under 2.0^26^.

1 $$ {\text{Al}}_{2} ({\text{SO}}_{4} )_{3} + 6{\text{H}}_{2} {\text{O}} \to 2{\text{Al}}({\text{OH}})_{3} + 3{{\text{SO}}_{4}}^{2 - } + 3{\text{H}}^{ + } $$

2 $$ {\text{FeSO}}_{4} + {\text{H}}_{2} {\text{O}} \to {\text{Fe}}({\text{OH}})_{3} + {{\text{SO}}_{4}}^{2 - } + 3{\text{H}}^{ + } $$

3 $$ {\text{FeS}}_{2} + 3.5{\text{O}}_{2} + {\text{H}}_{2} {\text{O}} \to {\text{Fe}}^{2 + } + 2{{\text{SO}}_{4}}^{2 + } + \, 2{\text{H}}^{ + } $$

The significant decrease observed in soil pH when going from CK-far to CK-near to the GP supported the scientific hypothesis that coal gangue affects the surrounding soil chemistry and continues to cause pollution 8 years later. The average pH value was low in GP (4.2 ± 0.53) to a level that is not suitable for most native plants, including *Carex rigescens, Artemisia argyi, *and* Conyza canadensis*. However, several native species, including *M. floridulus* and *H. punctata*, displayed strong acid resistance and were present in these plots.

After 8 years of natural succession, the abandoned mine areas had still not recovered to its natural state in terms of either soil chemical conditions or plant species composition.

Excess sulfur in the soil has been one of the main contributors to the altered environmental state of the study area.

### Sulfur (S) levels

In the study area (GP), S levels (1.03%) were over three times greater than in the plot 100 m away (CK-near; 0.30%) and 19 times greater than in the distant undisturbed plot (CK-far; 0.05%). In short, S levels varied significantly with coal gangue disturbance, and correlated negatively with both pH and plant species richness.

Acidification seriously affects soil health quality. Studies have shown that acidification leads to an increase in exchangeable acid and aluminum in soil, which is the main reason why acidified soil harms crop production^27^. The S content in natural soil is generally 0.1–1%, and plants get most of their S from the soil^28^. In grassland soils, exogenous sulfur input is one of the leading causes of acidification. Though sulfide is an important driver of acidification, sulfate is not an acid-producing ion. These sulfur inputs enhance the availability of nitrogen and micronutrients, including iron, manganese, copper, zinc and boron. They improve the synergetic uptake of nitrogen and phosphorus by plants, and thus they generally increase plant uptake of metallic micronutrients^28^.

**Figure 3:**
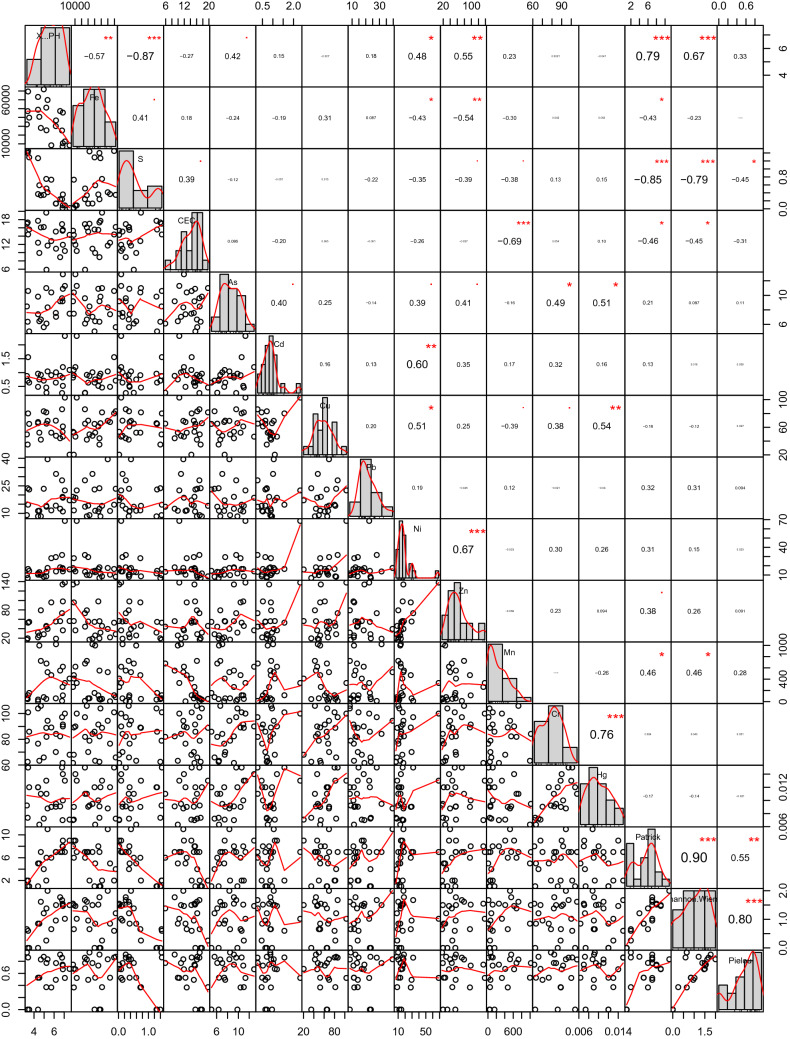
Pearson relationship between different soil properties across disturbed and undisturbed plots. n = 22, i.e. *p < 0.1; **p < 0.05; ***p < 0.01.

When pooled, all the data collected shows a strong negative Pearson relationship between sulfur and pH (R = − 0.868, n = 22, p < 0.001) (Fig. 3). A 50-year study of soil sulfur balance and organic carbon sink monitoring in grassland ecosystems in Northern Ireland showed that exogenous addition of sulfur is one of the most effective ways to cause soil and even groundwater acidification. It was shown that adding 1–3 t hm^−2^ sulfur to soil can lower the pH from 6–7 to below 3^29^.

The excessive amounts of S in disturbed soils is the main cause of soil acidification. Soil S content was found to decline as distance from the gangue heap increased. This indicates that sulfur excess is one of the main negative side effects from gangue leaching.

### Iron (Fe) levels

Fe levels in the soil also tended to be above the norm, but not to the same extent as S. Fe levels were high in both abandoned mine plots (GP) and CK-near, but levels were significantly lower in CK-far. Generally, greater than 4,000 mg kg^−1^ Fe in soil starts to be excessive.

It is possible that the iron was so high in the GP because (1) coal gangue contains large amounts of iron; and (2) acidic soil prevents iron absorption by plants. In the typical grassland in Inner Mongolia, studies have shown that the uptake of manganese by plants was greatly increased in acidified soil, while the absorption of iron was inhibited^30^. Fe levels remained high around the abandoned mine, which may suggest that iron degrades more slowly in natural processes^31^. High levels of iron may also be related to original Fe values, or to microbial catabolism^32^.

A case study at the Huainan coalfield in China^33^ indicated that trace elements (Mn, Cr, Ni, Zn, As and Cu) can pose serious environmental risks to the ecosystem. However, our results did not demonstrate a difference in heavy metal levels between CK-far and CK-near plots.

### Plant characteristics

Coal gangue significantly affected the distribution of plants. In the abandoned mine plots within the coal gangue (GP) both the abundance and richness of species were significantly lower than in the control plots (CK-near and CK-far). The adaptability of *M. floridulus* to acidic soil was stronger than for other plants, and so it became the dominant species in light of the acidic sulfur and high concentrations of iron. It was found that in the CK-near plots, which had an intermediate pH and sulfur level relative to the other plots, *H. punctata* grew well and become the dominant species in three out of six plots, even though these plants did not have the adaptability of *M. floridulus*, Of the native species, *H. punctata* grew the best in the disturbed areas. though *M. floridulus* adapted extremely well to the acidic soils with high S and Fe levels.

## Conclusion

Soil acidification and increased soil sulfur and iron content were well maintained in the coal gangue dump area after 8 years of natural recovery. The oxidation of sulfide and the acid mine drainage might be responsible for lowering the surrounding soil pH, though sulfur degrades fairly easily by natural processes, as shown by the 73% reduction in sulfur levels in the plot only 100 m away from the coal gangue. In contrast, Fe is not as easily degraded by natural processes. Despite the harsh conditions, native plant *M. floridulus* grew well in the strongly acidic coal gangue and became the dominant species in these disturbed areas. This highlights their potential value in helping to improve the soil environment of remaining gangue in Guizhou province, China. *M. floridulus* does not demonstrate the same dominance in undisturbed areas, which only further supports its potential value for treating abandoned coal gangue because it is not expected to alter the landscape long-term.

## Materials and methods

### Study area

The research was conducted in Huaxi District (106°27′–106°52E, 26°11′–26°34′) in the middle of Guizhou province. Huaxi District is located on the eastern slope of the Yunnan–Guizhou Plateau and has a subtropical monsoon humid climate, though it is also affected by its high altitude (the average altitude is 999 m), being cool in summer and warm in winter. The vertical decline rate of high temperatures is 0.6 °C/100 m in summer and only 0.4 °C/100 m in winter. The average annual temperature in the region is 14.9 °C, with 23.3 °C as the average highest temperature in July, and 4.7 °C as the average lowest temperature in January. The average number of days in January below 0 °C is 10.5 days, and the average number of days in July above 30 °C is 5.5 days. The average precipitation is abundant, 1,178.1 mm. July has the most precipitation. Huaxi district tends to have 75 days of spring, 89 days of summer, 68 days of autumn, and 133 days of winter, based on the average daily temperature (> 20 °C is summer, 10–20 °C s spring, 20–10 °C in autumn, and < 10 °C is winter). The annual frost-free period lasts 285 days. The average annual relative humidity is 81%. The average annual sunshine duration is 1,274.2 h, and the relative sunshine duration is 29%^34^.

The soil in the study area is zonal yellow soil, which is influenced by geological structure and lithology. The main agrotypes are yellow soil, lime soil and purple soil. The study area was located in a typical subtropical long-green broad-leaved forest vegetation belt, but the original vegetation has mostly disappeared, leaving behind only some typical tree species, such as *Viburnum henryi, Cinnamomum bodinieri*, etc. Introduced vegetation is mainly composed of Chinese *fir* and *Pinus massoniana *Lamb. plantations. The forest coverage rate of the whole region is at 48.38%. The study area is home to more than 120 families of plants and more than 1,000 species, including rare trees such as *Emmenopterys henryi, Hemlock, Zelkova schneideriana, Keteleeria fortunei* and so on.

The mineral resources in the area are mainly former coal mines, with proven existing reserves of 310 million tons and recoverable reserves of 110 million tons. Before 2011, coal mines were widely distributed throughout the district and the coal gangue they produced was piled up outside each. With the strengthened environmental protection efforts after 2011, most of the coal mines were closed, but the coal gangue remains.

### Field sampling

The three different selected coal mines (GP 1, 2 and 3) produced the same annual output of 90,000 tons in their average operational year. The coal gangue was stacked along the slope of the coal hole at each site. The area where the acid mine drainage possibly flowed from the abandoned mine was excluded from the selected plots. Above, in the center of and below these gangue dumps at each abandoned mine, a 2 m × 2 m plot was demarcated for vegetative investigation and sampling. Species, plant height, percent coverage and plant numbers were surveyed and recorded. Coal gangue and soil samples were collected (0–20 cm) using a soil auger (10 cm) with the five-point sampling method. Litter covering the sites was removed before sampling. Then, the five samples from the 2 m × 2 m plots were mixed into one sample for each plot. To homogenize the soil material, the samples were passed through a 2-mm sieve, which also removed live roots, mycorrhizal mycelia and coarse plant remnants. The soil samples were taken to the laboratory and divided into three subsamples. The subsamples for chemical analysis were air dried through a 0.15 mm sieve and stored at room temperature for the chemical analyses, and the subsamples for pH were passed through a 2-mm sieve.

**Figure 4:**
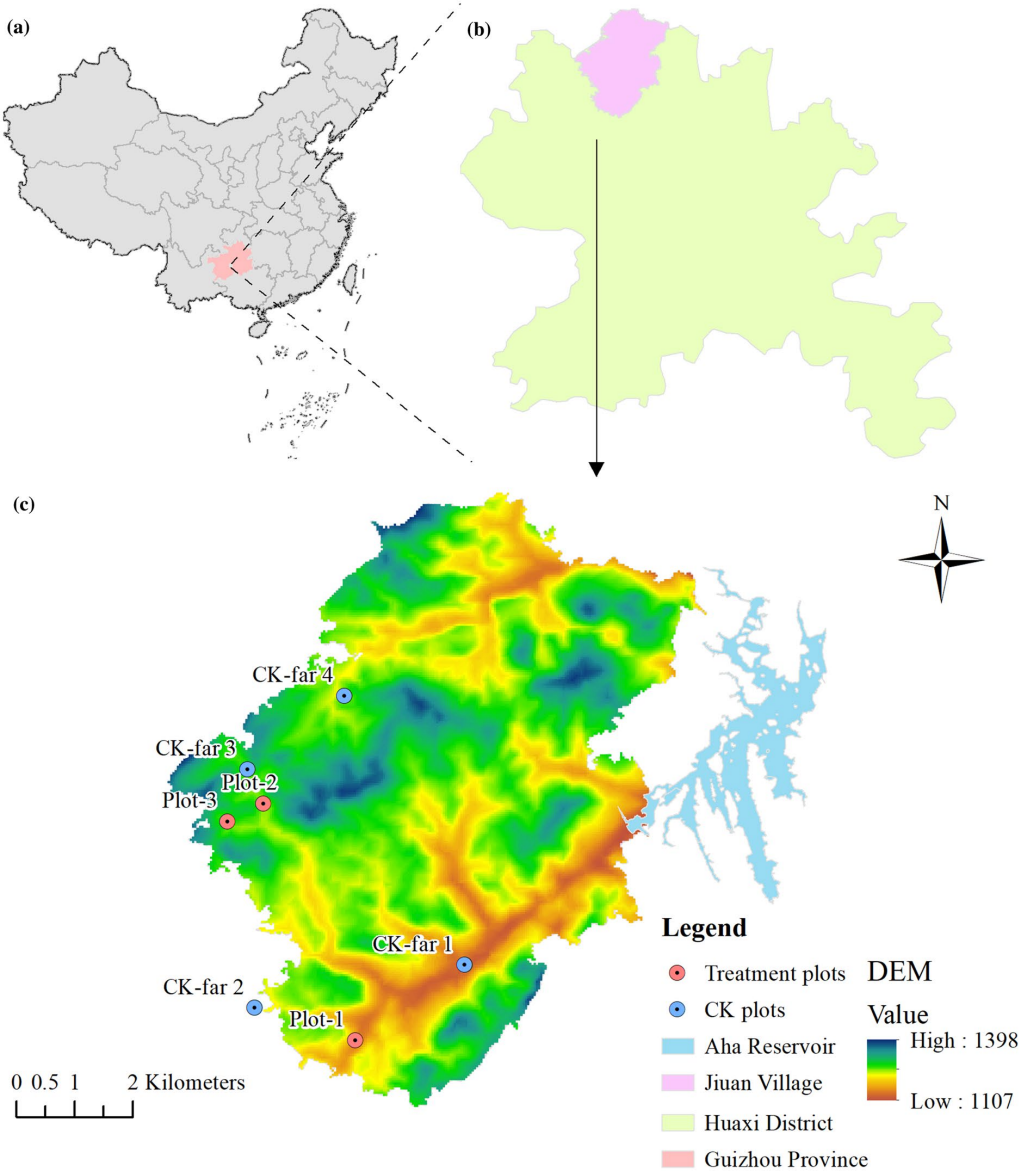
The geographical location information of the study area and distribution of sampling sites (ArcGIS Desktop. 10.3. ESRI, California, US. https://desktop.arcgis.com).

In contrast, three plots 100 m away from each gangue were selected as control areas (CK-near). The same plot plant survey and soil sample collection methods were conducted in the CK-near plots. Finally, an additional control group was surveyed to represent unmined land in a natural state. Four control plots (CK-far) of forest, farmland and lakeside that were distant from any coal mine were chosen, and then the 2 m × 2 m plot vegetation surveys and soil sampling were conducted there too (Table 3 and Fig. 4).

### Laboratory analysis

The levels of heavy metals (Cd, Cr, Cu, Ni, Zn, Mn, Pb) and As in the soil was tested using the national standard of China (HJ803-2016)^35^, where all samples are acid digested following a two-step digestion method that helps retain volatile elements. This consists of a “aqua regia” extraction followed by acid digestion of the residue. The resulting solution was then analyzed using an inductively coupled plasma mass spectrometer (ICP-MS, 7500a; Agilent China) for major and selected trace elements.

Iron levels in soil were determined using the national standard also (HJ 804-2016)^35^: the soil samples were air-dried, grinded and sifted to 2 mm. 10.0 g samples (accurate to 0.01 g) were placed in a 100 mL triangular bottle and 20.0 mL extract liquid (c (TEA) = 0.1 mol/L, c (CaCl_2_) = 0.01 mol/L, c (DTPA) = 0.005mol /L); pH = 7.3) was added. Then it was shaken at 20 °C ± 2 °C at a speed of 160–200 r/min for 2 h. The extraction solution was then slowly poured into the centrifuge tube and centrifuged for 10 min. The supernatant was isolated within 48 h after gravity filtration using medium-speed quantitative filter paper. A blank control supernatant was also made at the same time.

Hg levels in soil were determined using the national standard (GB/T 17136-1997): 0.5 g soil samples were wetted with a small amount of distilled water in a 150 mL volumetric flask. Then 10 mL of HNO_3_ and H_2_SO_4_ solution were added (1:1). After the intense reactions stopped, 10 mL of distilled water were added, and then 10 mL 0.02 g/mL K_2_MnSO_4_ solution. An electric heating plate was used to slowly heat the solution to near boiling, and this state was maintained for 30–60 min, being sure to add K_2_MnSO_4_ solution when the purple dissipated to ensure that K_2_MnSO_4_ was still present. Then the solution was cooled by shaking while adding 0.2 g/mL of hydroxylamine hydrochloride solution until the purple color of K_2_MnSO_4_ faded. And then tested with a cold atomic absorption mercury meter (F732-V; Hua Guang, Shanghai).

The soil sulfur concentrations were measured directly from a subset of air-dried samples using an elemental analyzer (Thermo Scientific FLASH 2000 CHNS/O; Waltham, MA, USA).

The soil pH values were determined using the standard method (HJ 962-2018), so the soil samples were air-dried, grinded and sifted to 2 mm, and then tested with a pH meter (Sartorius PB-10).

Determination of cation exchange capacity in soils were determined using the standard procedure (LY/T 1243-1999) with a centrifuge (TG-16W; Jinan Xinbei, China).

### Data analysis

The plant biodiversity indices were calculated as follows:

Shannon–Wiener index:

$$ H^{\prime} = - \mathop \sum {{P_{i} }} \ln P_{i} $$

Pielou index:

$$ E = \frac{{H^{\prime} }}{\ln S} $$

Important value:

$$ {\text{IV}} = \left( {{\text{Relative}}\;{\text{ plant}}\;{\text{ density}} + {\text{ Relative }}\;{\text{coverage}}\;{\text{ of }}\;{\text{plants}} + {\text{ Relative }}\;{\text{frequency}}} \right) \times 100/3 $$

$$ P_{i} = N_{i} /N $$

*Ni*: the important value of species i; *N*: the sum of the important values of all species in the plots; S: the number of species in the plot.

SPSS 20.0 (IBM, Chicago, USA) and R^36^ were used for all statistical analyses. ArcGIS 10.3 was used to make the distribution of sampling sites. Each plot was considered an experimental unit, and the replicated data was averaged among the plot types prior to analysis. Prior to performing ANOVA, all variables were checked for normality (Kolmogorov Smirnov test) and homogeneity (Levene's test). Then ANOVA analysis was performed. All results are reported as the mean value ± standard error. We considered p < 0.05 to be statistically significant. To examine the relationship between soil chemical variables, data from 22 independent plots was pooled and Pearson relationships were examined using the “Performance Analytics” package in R.

## Supplementary information

Supplementary information.
